# Antiviral activity of Brazilian Green Propolis extract against SARS-CoV-2 (Severe Acute Respiratory Syndrome - Coronavirus 2) infection: case report and review

**DOI:** 10.6061/clinics/2021/e2357

**Published:** 2021-01-18

**Authors:** Ana C. Fiorini, Carla A. Scorza, Antonio-Carlos G. de Almeida, Marcelo C.M. Fonseca, Josef Finsterer, Fernando L.A. Fonseca, Fulvio A. Scorza

**Affiliations:** I Departamento de Fonoaudiologia. Escola Paulista de Medicina / Universidade Federal de Sao Paulo (EPM/UNIFESP), Sao Paulo, SP, BR. Programa de Estudos Pos-Graduado em Fonoaudiologia, Pontificia Universidade Catolica de Sao Paulo (PUC-SP), Sao Paulo, SP, BR; II Centro de Neurociencias e Saude da Mulher “Professor Geraldo Rodrigues de Lima.” Escola Paulista de Medicina / Universidade Federal de Sao Paulo (EPM/UNIFESP), Sao Paulo, SP, BR; III Disciplina de Neurociencia. Escola Paulista de Medicina / Universidade Federal de Sao Paulo (EPM/UNIFESP), Sao Paulo, SP, BR; IV Laboratorio de Neurociencia Experimental e Computacional, Departamento de Engenharia de Biossistemas, Universidade Federal de Sao Joao del-Rei (UFSJ), Minas Gerais, MG, BR; V Departamento de Ginecologia. Nucleo de Avaliacao de Tecnologias em Saude. Escola Paulista de Medicina / Universidade Federal de Sao Paulo (EPM/UNIFESP), Sao Paulo, SP, BR; VI Krankenanstalt Rudolfstiftung, Messerli Institute, Vienna, Austria; VII Departamento de Patologia e Analises Clinicas, Programa de Pos-graduacao em Ciencias da Saude da FMABC, Santo André, SP, BR; VIII Departamento de Ciencias Farmaceuticas. Universidade Federal de Sao Paulo (UNIFESP), Diadema, SP, BR

Coronavirus disease (COVID-19) is caused by severe acute respiratory syndrome coronavirus 2 (SARS-CoV-2) (1). This novel coronavirus, which was first reported in Wuhan city, China, in late December 2019 and subsequently spreaded rapidly to all provinces of China and then worldwide, has had a devastating effect on global public health (2). As SARS-CoV-2 is highly contagious, the number of global infections has continuously increased since the first confirmed case a SARS-CoV-2 infection. The disease is often transmitted to the patient’s family members and medical staff (1). Clinically, 85% of infected patients have subclinical or mild disease. However, compared to flu, SARS-CoV-2 causes respiratory complications (e.g., severe pneumonia and acute respiratory distress syndrome [ground-glass opacities] and interstitial pneumonia) more easily, that is, in 10-15% of cases (2,3). In addition, 5% of infected patients require admission to the intensive care unit (2). Among the clinical manifestations of COVID-19, mortality is estimated at 0.7-7% (2,3). Although specific treatment for patients with COVID-19 is currently unavailable, there has been a consistent amendement in the clinical treatment plan (1). Numerous therapies, such as supportive intervention and the use immunomodulatory agents, antiviral therapy, antibiotics, anti-malarial agents, convalescent plasma transfusion, and blood transfusion-related technologies, have been adopted in clinical practice (1). In parallel, several translational studies have been conducted for vaccine development worldwide to combat SARS-CoV-2 infection (4). Additionally, a series of complementary treatments using medicinal plants and pure natural molecules isolated from plants have shown an important inhibitory antiviral activity against SARS-CoV-2 (5). From this clinical perspective, propolis can be used as a possible complementary treatment for patients with SARS-CoV-2.

Natural products have been increasingly applied for healing, actively attracting the attention of biomedical research and technology (6-8). However, physicians and researchers are not yet prepared to deepen their knowledge about biomimicry, probably because of a lack of exposure to biomimicry during training (8,9). Biomimicry is an area of science that deals with nature's models and emulation of these forms, processes, systems, and strategies to solve human health problems (6,7,9,10). Experts are clear in stating that biomimicry is based not only on what we can extract from organisms and their ecosystems but also on what we can learn from them (7-9). Following this line of reasoning, bee products have been applied in both traditional and modern medicine in recent years (11). In these lines, propolis and its extracts have had positive repercussions in the treatment of several diseases owing to its mechanisms related to pharmacological activities (11). The term “propolis” has a Greek origin and means defense for (“pro”) the community (“polis”), which refers to beehive (11,12). Propolis is defined as a balsamic and resinous product secreted by bees and composed of a mixture of 50% plant resins, 30% waxes, 10% essential and aromatic oils, 5% pollen, and 5% other organic substances (13,14). Although propolis functions as a sealant for holes and cracks in the beehive, its primary functions are related to smoothening the inner surface of the beehive, thereby maintaining the hive's internal temperature (35°C) and preventing weathering and invasion by predators (11).

Interestingly, the biological activities of propolis are attributed to a variety of chemical constituents such as phenolic acids, phenolic acid esters, flavonoids, terpenoids, artepillin C, caffeic acid, chrysin, galangin, quercetin, apigenin, kaempferol, pinobanksin 5-methyl ether, pinobanksin, pinocembrin, and pinobanksin 3-acetate (13). Propolis has a wide range of applications because of its pharmacological properties including antioxidant, antimicrobial, antiviral, anti-parasitic, anti-neoplastic, immunomodulatory, anti-inflammatory, and hepatoprotective properties (8,14,15). In the general context, the various therapeutic functions of propolis justify its potential use in the development of viable products useful to human health (8,14). Thus, some research proposals have exciting perspectives, especially regarding the use of propolis for treating individuals infected with SARS-CoV-2.

Over the past decade, several translational studies have demonstrated that propolis extract (PE) collected from temperate climate exerts potent and broad-spectrum antiviral activity against a diverse panel of viruses such as herpes simplex virus type 1 (HSV-1), herpes simplex virus type 2 (HSV-2), influenza virus types A and B, and human immunodeficiency virus (HIV) (16). In 2016, Mazia and colleagues evaluated the *in vitro* anti-HSV-1 activity of Brazilian green PE and its efficacy against cutaneous HSV-1 infection in a mouse model (17). In brief, the authors demonstrated the *in vitro* protective effect of Brazilian green PE against HSV-1, thereby confirming previously published data on propolis collected from other sources (17). Concerning HSV-2, an interesting study investigated the antiviral activities of propolis samples against HSV-1 and HSV-2 in the HEp-2 cell line and the possible synergistic effects of propolis with acyclovir against these viruses (18). Propolis extracts demonstrated significant antiviral effects compared with acyclovir. In particular, the antiviral activity exerted by propolis and acyclovir in combination against HSV-1 and HSV-2 was more pronounced than that exerted by acyclovir alone (18).

Furthermore, *in vitro* and *in vivo* studies verified that ethanol extracts of Brazilian propolis exhibited antiviral activity against the influenza virus (19). Brazilian propolis exhibits antiviral activity against influenza virus and ameliorates influenza symptoms in mice, suggesting that propolis may be a possible candidate for use as an anti-influenza dietary supplement in humans (19). Regarding HIV, studies conducted since the 1990s have shown that propolis suppresses HIV-1 replication and modulates *in vitro* immune responses (20). Moreover, the possible antiviral (infectious bursal disease virus and reovirus) and antimicrobial (*Staphylococcus aureus*, *Escherichia coli*, and *Candida albicans*) activities of propolis extracts were investigated. Briefly, Dakahlia propolis (typical poplar propolis from the East Nile Delta) has shown a high antiviral activity against infectious bursal disease virus, high antibacterial activity against *Escherichia coli*, and high antifungal activity against *Candida albicans* (21). Despite these relevant and positive data regarding propolis, the precise mechanisms underlying its antiviral activity are unknown; however, it is likely that propolis inhibits the entry of pathogens into the cells and disrupts the viral replication machinery (8,16). Unfortunately, studies evaluating propolis-mediated antiviral effects against SARS-CoV-2 are scarce, but the current results are very encouraging (8,16). In the early 1990s, propolis flavonoids were shown to reduce of the infectivity and replication of some herpesvirus, adenovirus, rotavirus, and coronavirus strains (8,16,22). One of the main active, high-efficiency flavonoids of propolis used for the treatment of SARS is quercetin (8,16,23). Quercetin in conjunction with vitamin C has shown aminopeptidase inhibitory activity, thereby interrupting the main proteases of SARS (8,16,23). Recently, a study based on simulations of molecular dynamics revealed a strong possibility that withanone (Wi-N) (active withanolides of Ashwagandha) and caffeic acid phenethyl ester (a bioactive ingredient of propolis) possess the potential to inhibit the functional activity of SARS-CoV-2 protease (an essential protein for virus survival) (24). Despite the crucial need for experimental and clinical investigations, the authors believe that the results of this study represent extraordinary therapeutic value for the management of COVID-19 (24).

To date, there are no proven effective therapies or vaccines for SARS-CoV-2 (25). Thus, identifying new therapeutic options is crucial to combat the disease (26). On the basis of this information, our research group evaluated a patient with COVID-19 who received Brazilian green PE. On June 13, 2020, a 52-year-old woman was admitted to our department with a 2-day history of headache, sore throat, and malaise. She had a blood pressure of 120/70 mmHg, pulse rate of 72 beats per min, respiratory rate of 18 rpm, body temperature of 36.7^o^C, and arterial oxygen saturation of 97% at room air. The patient was alert and coherent, and physical examination did not reveal wheezing on chest auscultation.

The patient had no past history of chronic diseases including kidney disease, hypertension, endocrine disorders, or neurological and musculoskeletal diseases. Moreover, she reported smoking but no alcohol intake. In addition, her routine blood test results were within the normal reference range at hospital admission. However, the reverse transcription-polymerase chain reaction (RT-PCR) assay for SARS-CoV-2 of a nasopharyngeal swab sample yielded a positive result. The patient was advised by the medical team to stay at home. At home, the patient maintained a healthy diet and adequate hydration. She also reported that she had started consuming the non-alcoholic preparation of Brazilian green propolis (EPP-AF^®^, Apis Flora Indl. Coml. Ltda, Ribeirão Preto, Brazil), at a dose of 45 drops three times a day for 14 days. After 12 days of treatment, the patient's general clinical condition improved significantly, and she recovered, with a negative RT-PCR test result. To the best of our knowledge, this is the first reported case indicating the therapeutic potential of propolis in SARS-CoV-2 infection. The relevance of this therapeutic intervention based on a single case report is debatable because the clinical effectiveness could have been influenced by several factors, including the patient's response to the use of other adjuvant therapies.

On the basis of these facts, what is the suggested course of action? First, it is clear that there are no medical therapies that can improve the outcomes of SARS-CoV-2-infected patients (27). To date, some pharmacological agents have demonstrated *in vitro* activity against the SARS-CoV-2 virus, and more than 300 active clinical trials are underway to identify effective medical treatments for this infection (27), including propolis therapy (28,29). Second, it must be borne in mind that patients with COVID-19 exhibit a spectrum of clinical manifestations ranging from being asymptomatic or having mild, self-limited constitutional symptoms to a hyperinflammatory state, followed by acute respiratory distress syndrome and death (30,31). Considering the current epidemiological picture, we cannot rule out that our 52-year-old patient had a mild form of the disease. However, clinical experience shows that older patients experience a more severe course and a greater need for hospitalization (32). Third, the use of propolis is an interesting challenge in the medical scientific world during this COVID-19 pandemic(8,16,28,29). Indeed, the possible therapeutic benefits reported for PEs against SARS-CoV-2 infection are an important focus in scientific research (8,16,28,29). It has been proposed that the immunomodulatory, antiviral, and anti-inflammatory effects of propolis and its chemical components are useful in the treatment of COVID-19 (16,28,29). As already established, angiotensin-converting enzyme 2 (ACE2) and transmembrane serine protease 2 (TMPRSS2) are the host cell receptors responsible for mediating infection by SARS-CoV-2 (33). This mechanism implies the overexpression of PAK1 (RAC/CDC42-activated kinase 1), the major “pathogenic” kinase whose abnormal activation causes a wide variety of diseases/disorders, including coronavirus-induced lung inflammation, fibrosis, and immune system suppression (28,34). In this context, it has been demonstrated that propolis components have inhibitory effects on the ACE2, TMPRSS2, and PAK1 signaling pathways, and its antiviral activity has been established in *in vitro* and *in vivo* studies (28). Another very important piece of information available is related to the role of inflammatory cytokines in the course and outcome of COVID-19 (35). In this sense, several translational studies clearly showed that propolis promoted the immunoregulation of pro-inflammatory cytokines such as interleukin (IL)-6, IL-1, and tumor necrosis factor-α (28). In general, these results open new avenues for the use of propolis as a natural co-adjuvant in the treatment of COVID-19.

Overall, there is a global effort to fighting this threat on several fronts in order to restore economic collapse, health systems, lifestyle habits, and safe living (36,37). Until the return to “normal life,” (26) preventive strategies and actions are needed reduce the transmission of the disease. These strategies include hand hygiene, the use of face masks, social distancing, and government-led lockdown of unnecessary activities to reduce the risk of disease transmission.

## References

[1] Li Y, Liu S, Zhang S, Ju Q, Zhang S, Yang Y (2020). Current treatment approaches for COVID-19 and the clinical value of transfusion-related technologies. Transfus Apher Sci.

[2] Di Pasquale G (2020). Coronavirus COVID-19: quali implicazioni per la Cardiologia? [COVID-19 coronavirus: what implications for Cardiology?]. G Ital Cardiol (Rome).

[3] Li T, Lu H, Zhang W (2020). Clinical observation and management of COVID-19 patients. Emerg Microbes Infect.

[4] Moore JP, Klasse PJ (2020). COVID-19 Vaccines: “Warp Speed” Needs Mind Melds, Not Warped Minds. J Virol.

[5] Orhan IE, Senol Deniz FS (2020). Natural Products as Potential Leads Against Coronaviruses: Could They be Encouraging Structural Models Against SARS-CoV-2?. Nat Prod Bioprospect.

[6] What is biomimicry? http://www.biomimicryinstitute.org/about-us/what-is-biomimicry.html.

[7] What do you mean by the term biomimicry? A conversation with Janine Benyus, author of biomimicry: innovation inspired by in nature.

[8] Scorza CA, Gonçalves VC, Scorza FA, Fiorini AC, de Almeida AG, Fonseca MCM (2020). Propolis and coronavirus disease 2019 (COVID-19): Lessons from nature. Complement Ther Clin Pract.

[9] Scorza FA, Cavalheiro EA (2010). Biomimicry: Applying design for nature to solve problems in epilepsy research. Epilepsy Behav.

[10] Benyus JM (2002). Biomimicry: Innovation Inspired by Nature.

[11] Pasupuleti VR, Sammugam L, Ramesh N, Gan SH (2017). Honey, Propolis, and Royal Jelly: A Comprehensive Review of Their Biological Actions and Health Benefits. Oxid Med Cell Longev.

[12] Castaldo S, Capasso F (2002). Propolis, an old remedy used in modern medicine. Fitoterapia.

[13] Huang S, Zhang CP, Wang K, Li GQ, Hu FL (2014). Recent advances in the chemical composition of propolis. Molecules.

[14] Santos LM, Fonseca MS, Sokolonski AR, Deegan KR, Araújo RP, Umsza-Guez MA (2020). Propolis: types, composition, biological activities, and veterinary product patent prospecting. J Sci Food Agric.

[15] Braakhuis A (2019). Evidence on the Health Benefits of Supplemental Propolis. Nutrients.

[16] Bachevski D, Damevska K, Simeonovski V, Dimova M (2020). Back to the basics: Propolis and COVID-19. Dermatol Ther.

[17] Mazia RS, de Araújo Pereira RR, de Francisco LM, Natali MR, Dias Filho BP, Nakamura CV (2016). Formulation and Evaluation of a Mucoadhesive Thermoresponsive System Containing Brazilian Green Propolis for the Treatment of Lesions Caused by Herpes Simplex Type I. J Pharm Sci.

[18] Yildirim A, Duran GG, Duran N, Jenedi K, Bolgul BS, Miraloglu M (2016). Antiviral Activity of Hatay Propolis Against Replication of Herpes Simplex Virus Type 1 and Type 2. Med Sci Monit.

[19] Shimizu T, Hino A, Tsutsumi A, Park YK, Watanabe W, Kurokawa M (2008). Anti-influenza virus activity of propolis *in vitro* and its efficacy against influenza infection in mice. Antivir Chem Chemother.

[20] Harish Z, Rubinstein A, Golodner M, Elmaliah M, Mizrachi Y (1997). Suppression of HIV-1 replication by propolis and its immunoregulatory effect. Drugs Exp Clin Res.

[21] Abd El Hady FK, Hegazi AG (2002). Egyptian propolis: 2. Chemical composition, antiviral and antimicrobial activities of East Nile Delta propolis. Z Naturforsch C J Biosci.

[22] Debiaggi M, Tateo F, Pagani L, Luini M, Romero E (1990). Effects of propolis flavonoids on virus infectivity and replication. Microbiologica.

[23] Syed S, Saleem A (2004). Severe Acute Respiratory Syndrome Epidemiology and Control. Lab Med.

[24] Kumar V, Dhanjal JK, Kaul SC, Wadhwa R, Sundar D (2020). Withanone and caffeic acid phenethyl ester are predicted to interact with main protease (Mpro) of SARS-CoV-2 and inhibit its activity. J Biomol Struct Dyn.

[25] Nabil A, Uto K, Elshemy MM, Soliman R, Hassan AA, Ebara M (2020). Current coronavirus (SARS-CoV-2) epidemiological, diagnostic and therapeutic approaches: An updated review until June 2020. EXCLI J.

[26] Adil MT, Rahman R, Whitelaw D, Jain V, Al-Taan O, Rashid F (2020). SARS-CoV-2 and the pandemic of COVID-19. Postgrad Med J.

[27] Sanders JM, Monogue ML, Jodlowski TZ, Cutrell JB (2020). Pharmacologic Treatments for Coronavirus Disease 2019 (COVID-19): A Review. JAMA.

[28] Berretta AA, Silveira MAD, Cóndor Capcha JM, De Jong D (2020). Propolis and its potential against SARS-CoV-2 infection mechanisms and COVID-19 disease: Running title: Propolis against SARS-CoV-2 infection and COVID-19. Biomed Pharmacother.

[29] Lima WG, Brito JCM, da Cruz Nizer WS (2020). Bee products as a source of promising therapeutic and chemoprophylaxis strategies against COVID-19 (SARS-CoV-2). Phytother Res.

[30] Blanco F, Matute H (2020). Diseases that resolve spontaneously can increase the belief that ineffective treatments work. Soc Sci Med.

[31] Afadi MAP, Silva CAAD (2020). The Challenging and Unpredictable Spectrum of Covid-19 in Children and Adolescents. Rev Paul Pediatr.

[32] Coronavirus disease 2019 (COVID-19) Centers for Disease Control and Prevention.

[33] Wu J, Deng W, Li S, Yang X (2020). Advances in research on ACE2 as a receptor for 2019-nCoV. Cell Mol Life Sci.

[34] Maruta H, He H (2020). PAK1-blockers: Potential Therapeutics against COVID-19. Med Drug Discov.

[35] Del Valle DM, Kim-Schulze S, Huang HH, Beckmann ND, Nirenberg S, Wang B (2020). An inflammatory cytokine signature predicts COVID-19 severity and survival. Nat Med.

[36] Laskar P, Yallapu MM, Chauhan SC (2020). “Tomorrow Never Dies”: Recent Advances in Diagnosis, Treatment, and Prevention Modalities against Coronavirus (COVID-19) amid Controversies. Diseases.

[37] Danielli S, Patria R, Donnelly P, Ashrafian H, Darzi A (2020). Economic interventions to ameliorate the impact of COVID-19 on the economy and health: an international comparison. J Public Health (Oxf).

